# 1-(1*H*-Imidazo[4,5-*f*][1,10]phenan­throlin-2-yl)naphthalen-2-ol

**DOI:** 10.1107/S1600536811005861

**Published:** 2011-02-26

**Authors:** Xiu-Yan Wang, Shuai Ma, Yu He

**Affiliations:** a College of Chemistry, Jilin Normal University, Siping 136000, People’s Republic of China, and Key Laboratory of Preparation and Applications of Environmentally Friendly Materials (Jilin Normal University), Ministry of Education, People’s Republic of China

## Abstract

In the title mol­ecule, C_23_H_14_N_4_O, the dihedral angle between the pyridine rings of the phenanthroline unit is 4.43 (8)° and the dihedral angle formed by the nine essentially planar [maximum deviation 0.0389 (16)Å] non-H atoms of the benzimidazole unit and the naphthalene ring system is 74.22 (5)°. In the crystal, mol­ecules are linked by inter­molecular N—H⋯N and O—H⋯N hydrogen bonds, forming a three-dimensional network.

## Related literature

For background to the coordination chemistry of 1,10-phenanthroline derivatives, see: Wang *et al.* (2010 ▶). For the synthetic procedure, see: Steck & Day (1943 ▶).
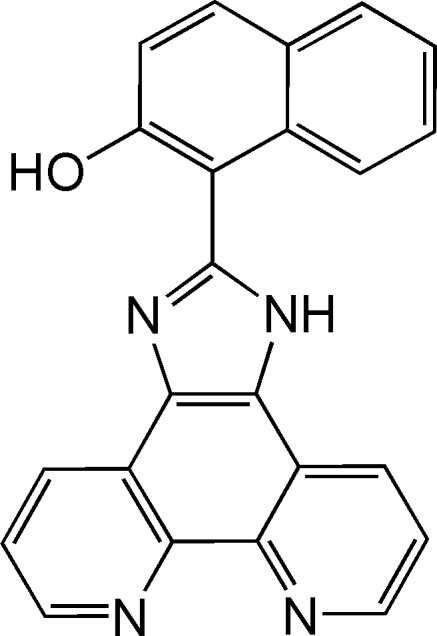


## Experimental

### 

#### Crystal data

C_23_H_14_N_4_O
*M* *_r_* = 362.38
Tetragonal, 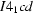

*a* = 22.5800 (4) Å
*c* = 13.7196 (5) Å
*V* = 6995.0 (3) Å^3^
*Z* = 16
Mo *K*α radiation
μ = 0.09 mm^−1^
*T* = 293 K
0.30 × 0.21 × 0.18 mm

#### Data collection

Bruker APEX diffractometer
Absorption correction: multi-scan (*SADABS*; Sheldrick, 1996 ▶) *T*_min_ = 0.41, *T*_max_ = 0.72
18374 measured reflections
3433 independent reflections
3153 reflections with *I* > 2σ(*I*)
*R*_int_ = 0.022

#### Refinement

*R*[*F* ^2^ > 2σ(*F* ^2^)] = 0.031
*wR*(*F* ^2^) = 0.081
*S* = 1.06
3433 reflections
253 parameters
1 restraint
H-atom parameters constrained
Δρ_max_ = 0.12 e Å^−3^
Δρ_min_ = −0.14 e Å^−3^
Absolute structure: Flack (1983 ▶), 1629 Friedel pairs
Flack parameter: 0.0 (13)

### 

Data collection: *SMART* (Bruker, 1997 ▶); cell refinement: *SAINT* (Bruker, 1999 ▶); data reduction: *SAINT*; program(s) used to solve structure: *SHELXS97* (Sheldrick, 2008 ▶); program(s) used to refine structure: *SHELXL97* (Sheldrick, 2008 ▶); molecular graphics: *SHELXTL* (Sheldrick, 2008 ▶); software used to prepare material for publication: *SHELXL97*.

## Supplementary Material

Crystal structure: contains datablocks global, I. DOI: 10.1107/S1600536811005861/lh5209sup1.cif

Structure factors: contains datablocks I. DOI: 10.1107/S1600536811005861/lh5209Isup2.hkl

Additional supplementary materials:  crystallographic information; 3D view; checkCIF report

## Figures and Tables

**Table 1:** Hydrogen-bond geometry (Å, °)

*D*—H⋯*A*	*D*—H	H⋯*A*	*D*⋯*A*	*D*—H⋯*A*
N4—H4⋯N2^i^	0.86	2.17	2.9361 (19)	149
N4—H4⋯N1^i^	0.86	2.50	3.191 (2)	138
O1—H1⋯N3^ii^	0.82	2.01	2.7203 (17)	145

Symmetry codes: (i) 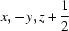; (ii) 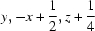.

**Fig. 1.**
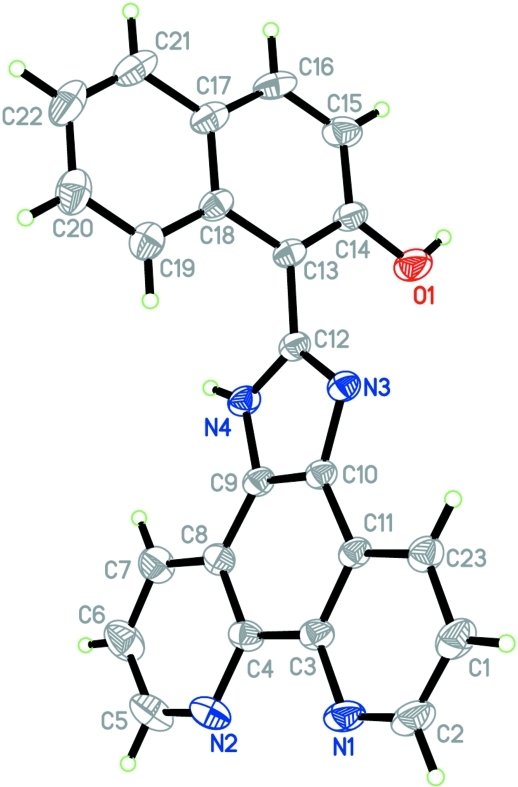
The molecular structure of of the title compound showing displacement ellipsoids drawn at the 30% probability.

## supplementary crystallographic information

### Comment

1,10-Phenanthroline and its derivatives, are potentially important chelating ligands with excellent coordinating abilities and have been extensively used to build supramolecular architectures (Wang *et al.*, 2010). We report herein the synthesis and crystal structure of the title compound

The molecular structure of the title compound is shown in Fig. 1. The dihedral angle between the pyridine rings of the phenanthroline unit [N2/C4-C8 and N1/C1/C2/C3/C11/C23] is 4.43 (8)Å and the dihedral angle formed by the nine essentially planar non-hydrogen atoms of the benzimidazole unit [C3/C4/C8-C12; maximum deviation 0.0389 (16)Å for C4] and the naphthalene ring system is 74.22 (5)°. In the crystal, molecules are linked by intermolecular N—H···N and O—H···N hydrogen bonds to form a three-dimensional network.

### Experimental

The title compound was synthesized according to the literature method of Steck & Day (1943). We carried out the following reaction but the unreacted title compound was found in the reaction vessel. A mixture of Bi(NO_3_)_3_^.^5H_2_O (0.5 mmol) and L (0.5 mmol) in 10 mL distilled water was heated at 463 K in a Teflon-lined stainless steel autoclave for three days. The reaction system was then slowly cooled to room temperature. Pale yellow crystals suitable for single crystal X-ray diffraction analysis were collected from the final reaction system.

### Refinement

All H atoms were positioned geometrically (N—H = 0.86, C—H = 0.93 and O—H = 0.82 Å ) and refined as riding, with U_iso_(H) = 1.2U_eq_(C,N) or U_iso_(H) = 1.5U_eq_(O).

### Crystal data

**Table:**

C_23_H_14_N_4_O	*D*_x_ = 1.376 Mg m^−^^3^
*M**_r_* = 362.38	Mo *K*α radiation, λ = 0.71073 Å
Tetragonal, *I*4_1_*c**d*	Cell parameters from 3433 reflections
Hall symbol: I 4bw -2c	θ = 1.8–26.0°
*a* = 22.5800 (4) Å	µ = 0.09 mm^−^^1^
*c* = 13.7196 (5) Å	*T* = 293 K
*V* = 6995.0 (3) Å^3^	Block, pale yellow
*Z* = 16	0.30 × 0.21 × 0.18 mm
*F*(000) = 3008	

### Data collection

**Table:**

Bruker APEX diffractometer	3433 independent reflections
Radiation source: fine-focus sealed tube	3153 reflections with *I* > 2σ(*I*)
graphite	*R*_int_ = 0.022
φ and ω scans	θ_max_ = 26.0°, θ_min_ = 1.8°
Absorption correction: multi-scan (*SADABS*; Sheldrick, 1996)	*h* = −27→27
*T*_min_ = 0.41, *T*_max_ = 0.72	*k* = −27→20
18374 measured reflections	*l* = −16→16

### Refinement

**Table:**

Refinement on *F*^2^	Secondary atom site location: difference Fourier map
Least-squares matrix: full	Hydrogen site location: inferred from neighbouring sites
*R*[*F*^2^ > 2σ(*F*^2^)] = 0.031	H-atom parameters constrained
*wR*(*F*^2^) = 0.081	*w* = 1/[σ^2^(*F*_o_^2^) + (0.0457*P*)^2^ + 1.1603*P*] where *P* = (*F*_o_^2^ + 2*F*_c_^2^)/3
*S* = 1.06	(Δ/σ)_max_ = 0.001
3433 reflections	Δρ_max_ = 0.12 e Å^−^^3^
253 parameters	Δρ_min_ = −0.14 e Å^−^^3^
1 restraint	Absolute structure: Flack (1983), 1629 Friedel pairs
Primary atom site location: structure-invariant direct methods	Flack parameter: 0.0 (13)

### Special details

**Table:**

Geometry. All esds (except the esd in the dihedral angle between two l.s. planes) are estimated using the full covariance matrix. The cell esds are taken into account individually in the estimation of esds in distances, angles and torsion angles; correlations between esds in cell parameters are only used when they are defined by crystal symmetry. An approximate (isotropic) treatment of cell esds is used for estimating esds involving l.s. planes.
Refinement. Refinement of F^2^ against ALL reflections. The weighted R-factor wR and goodness of fit S are based on F^2^, conventional R-factors R are based on F, with F set to zero for negative F^2^. The threshold expression of F^2^ > 2sigma(F^2^) is used only for calculating R-factors(gt) etc. and is not relevant to the choice of reflections for refinement. R-factors based on F^2^ are statistically about twice as large as those based on F, and R- factors based on ALL data will be even larger.

### Fractional atomic coordinates and isotropic or equivalent isotropic displacement parameters (Å^2^)

**Table:**

	*x*	*y*	*z*	*U*_iso_*/*U*_eq_	
N4	0.15231 (6)	0.04557 (6)	0.26423 (9)	0.0422 (3)	
H4	0.1345	0.0224	0.3045	0.051*	
O1	0.15580 (7)	0.17926 (6)	0.40232 (9)	0.0690 (4)	
H1	0.1466	0.2049	0.4418	0.104*	
N1	0.14620 (7)	0.08057 (7)	−0.13144 (9)	0.0534 (4)	
C14	0.19348 (7)	0.14069 (7)	0.44439 (12)	0.0468 (4)	
N2	0.08332 (6)	−0.01202 (7)	−0.06297 (11)	0.0569 (4)	
C4	0.11269 (7)	0.02173 (7)	0.00293 (11)	0.0442 (4)	
C12	0.19309 (6)	0.08738 (6)	0.28685 (10)	0.0389 (3)	
N3	0.21230 (5)	0.11616 (5)	0.20892 (9)	0.0398 (3)	
C11	0.18392 (7)	0.10501 (6)	0.02914 (10)	0.0386 (3)	
C3	0.14849 (6)	0.07058 (7)	−0.03403 (10)	0.0421 (3)	
C18	0.25212 (6)	0.05078 (7)	0.42951 (11)	0.0437 (3)	
C13	0.21381 (7)	0.09461 (7)	0.38867 (11)	0.0410 (3)	
C20	0.31013 (8)	−0.03905 (8)	0.41619 (17)	0.0641 (5)	
H20	0.3238	−0.0707	0.3791	0.077*	
C9	0.14476 (6)	0.04705 (6)	0.16521 (10)	0.0390 (3)	
C1	0.21610 (9)	0.15939 (8)	−0.11023 (13)	0.0585 (5)	
H1A	0.2389	0.1889	−0.1391	0.070*	
C19	0.27385 (7)	0.00224 (8)	0.37510 (13)	0.0516 (4)	
H19	0.2632	−0.0016	0.3099	0.062*	
C21	0.30698 (8)	0.01212 (10)	0.56875 (15)	0.0677 (6)	
H21	0.3185	0.0151	0.6337	0.081*	
C15	0.21017 (8)	0.14513 (9)	0.54344 (13)	0.0576 (4)	
H15	0.1961	0.1762	0.5815	0.069*	
C8	0.10968 (7)	0.01000 (7)	0.10380 (11)	0.0422 (3)	
C10	0.18153 (6)	0.09101 (6)	0.13142 (10)	0.0373 (3)	
C7	0.07478 (8)	−0.03699 (8)	0.13643 (14)	0.0582 (4)	
H7	0.0721	−0.0458	0.2025	0.070*	
C17	0.26900 (7)	0.05588 (8)	0.52860 (13)	0.0516 (4)	
C2	0.17893 (9)	0.12442 (9)	−0.16609 (13)	0.0613 (5)	
H2	0.1768	0.1323	−0.2325	0.074*	
C16	0.24695 (8)	0.10373 (10)	0.58300 (13)	0.0601 (5)	
H16	0.2578	0.1073	0.6481	0.072*	
C5	0.05054 (10)	−0.05570 (10)	−0.02947 (17)	0.0720 (6)	
H5	0.0300	−0.0787	−0.0744	0.086*	
C6	0.04461 (9)	−0.06976 (9)	0.06877 (17)	0.0729 (6)	
H6	0.0205	−0.1010	0.0882	0.087*	
C23	0.21879 (8)	0.14991 (7)	−0.01156 (12)	0.0473 (4)	
H23	0.2433	0.1729	0.0276	0.057*	
C22	0.32682 (9)	−0.03397 (10)	0.51413 (19)	0.0726 (6)	
H22	0.3516	−0.0623	0.5419	0.087*	

### Atomic displacement parameters (Å^2^)

**Table:**

	*U*^11^	*U*^22^	*U*^33^	*U*^12^	*U*^13^	*U*^23^
N4	0.0482 (7)	0.0475 (7)	0.0308 (6)	−0.0029 (6)	0.0025 (5)	0.0074 (5)
O1	0.0956 (10)	0.0655 (8)	0.0461 (7)	0.0325 (7)	−0.0183 (7)	−0.0127 (6)
N1	0.0634 (9)	0.0683 (9)	0.0286 (6)	0.0102 (7)	0.0019 (6)	−0.0026 (6)
C14	0.0506 (8)	0.0539 (9)	0.0358 (8)	0.0075 (7)	−0.0057 (7)	0.0015 (7)
N2	0.0590 (8)	0.0651 (9)	0.0467 (8)	−0.0034 (7)	0.0024 (7)	−0.0196 (7)
C4	0.0427 (8)	0.0511 (9)	0.0387 (8)	0.0058 (7)	0.0031 (6)	−0.0095 (7)
C12	0.0418 (7)	0.0431 (7)	0.0318 (7)	0.0050 (6)	−0.0003 (6)	0.0024 (6)
N3	0.0430 (6)	0.0443 (6)	0.0321 (6)	0.0014 (5)	0.0008 (5)	0.0029 (5)
C11	0.0424 (7)	0.0433 (8)	0.0303 (7)	0.0072 (6)	0.0043 (6)	0.0024 (6)
C3	0.0438 (8)	0.0503 (8)	0.0321 (8)	0.0092 (6)	0.0043 (6)	−0.0041 (6)
C18	0.0381 (7)	0.0521 (8)	0.0409 (8)	−0.0018 (6)	−0.0005 (6)	0.0117 (7)
C13	0.0442 (8)	0.0489 (8)	0.0299 (7)	0.0002 (6)	−0.0014 (6)	0.0058 (6)
C20	0.0497 (9)	0.0542 (10)	0.0883 (15)	0.0043 (7)	0.0029 (10)	0.0120 (10)
C9	0.0421 (7)	0.0444 (8)	0.0304 (7)	0.0022 (6)	0.0033 (6)	0.0036 (6)
C1	0.0691 (11)	0.0645 (11)	0.0417 (9)	0.0023 (8)	0.0123 (8)	0.0152 (8)
C19	0.0458 (8)	0.0523 (9)	0.0566 (10)	0.0011 (7)	0.0016 (7)	0.0069 (8)
C21	0.0561 (10)	0.0880 (15)	0.0590 (11)	−0.0003 (10)	−0.0157 (9)	0.0290 (10)
C15	0.0649 (10)	0.0704 (11)	0.0375 (9)	0.0043 (8)	−0.0026 (8)	−0.0084 (8)
C8	0.0417 (8)	0.0443 (8)	0.0405 (8)	0.0017 (6)	0.0039 (6)	−0.0036 (6)
C10	0.0398 (7)	0.0424 (8)	0.0297 (7)	0.0029 (6)	0.0016 (6)	0.0008 (6)
C7	0.0633 (11)	0.0557 (10)	0.0557 (11)	−0.0100 (8)	0.0084 (8)	0.0002 (8)
C17	0.0441 (8)	0.0673 (10)	0.0433 (9)	−0.0035 (7)	−0.0061 (7)	0.0154 (8)
C2	0.0767 (12)	0.0770 (12)	0.0302 (8)	0.0113 (10)	0.0063 (8)	0.0087 (8)
C16	0.0627 (10)	0.0864 (13)	0.0314 (7)	−0.0018 (10)	−0.0115 (8)	0.0070 (8)
C5	0.0764 (13)	0.0718 (12)	0.0679 (13)	−0.0183 (10)	0.0033 (11)	−0.0268 (11)
C6	0.0809 (13)	0.0620 (12)	0.0756 (15)	−0.0267 (10)	0.0108 (11)	−0.0115 (10)
C23	0.0529 (9)	0.0505 (9)	0.0387 (8)	0.0023 (7)	0.0061 (7)	0.0043 (7)
C22	0.0569 (11)	0.0678 (13)	0.0932 (16)	0.0089 (9)	−0.0099 (11)	0.0312 (12)

### Geometric parameters (Å, °)

**Table:**

N4—C12	1.3548 (19)	C20—H20	0.9300
N4—C9	1.3697 (19)	C9—C10	1.375 (2)
N4—H4	0.8600	C9—C8	1.427 (2)
O1—C14	1.3476 (18)	C1—C23	1.372 (2)
O1—H1	0.8200	C1—C2	1.384 (3)
N1—C2	1.324 (2)	C1—H1A	0.9300
N1—C3	1.3563 (19)	C19—H19	0.9300
C14—C13	1.370 (2)	C21—C22	1.358 (3)
C14—C15	1.414 (2)	C21—C17	1.420 (2)
N2—C5	1.316 (3)	C21—H21	0.9300
N2—C4	1.356 (2)	C15—C16	1.363 (3)
C4—C8	1.411 (2)	C15—H15	0.9300
C4—C3	1.459 (2)	C8—C7	1.395 (2)
C12—N3	1.3241 (18)	C7—C6	1.369 (3)
C12—C13	1.482 (2)	C7—H7	0.9300
N3—C10	1.3912 (19)	C17—C16	1.404 (3)
C11—C23	1.400 (2)	C2—H2	0.9300
C11—C3	1.413 (2)	C16—H16	0.9300
C11—C10	1.4394 (19)	C5—C6	1.391 (3)
C18—C19	1.414 (2)	C5—H5	0.9300
C18—C17	1.417 (2)	C6—H6	0.9300
C18—C13	1.429 (2)	C23—H23	0.9300
C20—C19	1.363 (3)	C22—H22	0.9300
C20—C22	1.400 (3)		
			
C12—N4—C9	107.15 (12)	C20—C19—H19	119.3
C12—N4—H4	126.4	C18—C19—H19	119.3
C9—N4—H4	126.4	C22—C21—C17	121.23 (19)
C14—O1—H1	109.5	C22—C21—H21	119.4
C2—N1—C3	117.19 (16)	C17—C21—H21	119.4
O1—C14—C13	117.60 (14)	C16—C15—C14	119.76 (17)
O1—C14—C15	122.28 (14)	C16—C15—H15	120.1
C13—C14—C15	120.07 (14)	C14—C15—H15	120.1
C5—N2—C4	117.62 (16)	C7—C8—C4	118.98 (16)
N2—C4—C8	121.68 (15)	C7—C8—C9	124.73 (15)
N2—C4—C3	117.67 (14)	C4—C8—C9	116.26 (14)
C8—C4—C3	120.64 (14)	C9—C10—N3	109.79 (12)
N3—C12—N4	112.31 (13)	C9—C10—C11	120.66 (13)
N3—C12—C13	127.13 (13)	N3—C10—C11	129.55 (13)
N4—C12—C13	120.47 (13)	C6—C7—C8	118.34 (18)
C12—N3—C10	104.67 (11)	C6—C7—H7	120.8
C23—C11—C3	118.18 (14)	C8—C7—H7	120.8
C23—C11—C10	124.68 (14)	C16—C17—C18	118.50 (15)
C3—C11—C10	117.14 (13)	C16—C17—C21	122.93 (18)
N1—C3—C11	122.32 (15)	C18—C17—C21	118.57 (18)
N1—C3—C4	116.56 (14)	N1—C2—C1	124.50 (16)
C11—C3—C4	121.11 (13)	N1—C2—H2	117.8
C19—C18—C17	118.45 (15)	C1—C2—H2	117.8
C19—C18—C13	122.67 (14)	C15—C16—C17	122.14 (16)
C17—C18—C13	118.88 (15)	C15—C16—H16	118.9
C14—C13—C18	120.65 (14)	C17—C16—H16	118.9
C14—C13—C12	120.25 (13)	N2—C5—C6	124.30 (18)
C18—C13—C12	118.97 (14)	N2—C5—H5	117.9
C19—C20—C22	120.18 (19)	C6—C5—H5	117.9
C19—C20—H20	119.9	C7—C6—C5	119.06 (18)
C22—C20—H20	119.9	C7—C6—H6	120.5
N4—C9—C10	106.07 (13)	C5—C6—H6	120.5
N4—C9—C8	129.82 (13)	C1—C23—C11	118.80 (17)
C10—C9—C8	124.02 (13)	C1—C23—H23	120.6
C23—C1—C2	118.97 (17)	C11—C23—H23	120.6
C23—C1—H1A	120.5	C21—C22—C20	120.22 (17)
C2—C1—H1A	120.5	C21—C22—H22	119.9
C20—C19—C18	121.35 (17)	C20—C22—H22	119.9

### Hydrogen-bond geometry (Å, °)

**Table:**

*D*—H···*A*	*D*—H	H···*A*	*D*···*A*	*D*—H···*A*
N4—H4···N2^i^	0.86	2.17	2.9361 (19)	149
N4—H4···N1^i^	0.86	2.50	3.191 (2)	138
O1—H1···N3^ii^	0.82	2.01	2.7203 (17)	145

Symmetry codes: (i) *x*, −*y*, *z*+1/2; (ii) *y*, −*x*+1/2, *z*+1/4.
